# A High-Efficiency Environmentally Friendly Polishing Slurry for K9 Glass Utilizing Cerium-Based Compounds

**DOI:** 10.3390/molecules30234546

**Published:** 2025-11-25

**Authors:** Shaoping Li, Rui Ye, Zhemin Zou, Zhaobo He, Kai Feng, Huidong Cui, Ruan Chi, Yukun Chen, Yue Luo

**Affiliations:** 1 School of Resources and Safety Engineering, Wuhan Institute of Technology, 693 Xiongchu Avenue, Wuhan 430073, China; lishaoping@hbsxsys.com; 2 Hubei Sinophorus Electronic Materials Co., Ltd., Xiaoting Avenue 66-3#, Yichang 443007, China; 3 Hubei Three Gorges Laboratory, 1 Ma Zongling Road, Yichang 443007, China

**Keywords:** K9 glass, chemical mechanical polishing, mixed abrasive, guanidine carbonate, sodium lauryl-6 carboxylate

## Abstract

Owing to the rapid advancements in optical and microsystem technologies, K9 glass is extensively utilized in the fabrication of high-precision optical components. Nevertheless, the intrinsic brittleness and elevated hardness of K9 glass, combined with the stringent demands of high-end optical systems for exceptional surface precision and minimal subsurface damage, present significant challenges for its chemical mechanical polishing (CMP) process. To overcome this challenge, we formulated a novel environmentally friendly and high-performance polishing slurry comprising cerium oxide (CeO_2_), aluminum oxide (Al_2_O_3_), guanidine carbonate (GC), and sodium laureth-6 carboxylate (SL-6C). The incorporation of a minor proportion of high-hardness Al_2_O_3_ abrasive particles significantly enhanced the mechanical friction within the polishing slurry, thereby markedly increasing the MRR. The judicious addition of GC facilitated the formation of a hydration layer on the glass substrate. The surfactant SL-6C modulated the surface charge of the abrasive particles through electrostatic and coordination interactions, which improved particle dispersion and mitigated agglomeration. This effect minimized the risk of surface scratching and enhanced interfacial lubrication, consequently reducing the energy required for the detachment of the reaction layer. CMP findings demonstrated that utilizing an optimized slurry formulation comprising 1 wt% CeO_2_, 0.05 wt% Al_2_O_3_, 0.2 wt% GC, and 0.2 wt% SL-6C yielded a surface roughness of K9 glass as low as 0.11 nm. Additionally, the MRR value reached 521.71 nm/min. Compared with the polishing slurry containing only CeO_2_, the MRR increased by 7 times. The observed synergistic interactions among Al_2_O_3_, GC, SL-6C, and CeO_2_ offered valuable insights for the advancement of high-performance CMP slurries.

## 1. Introduction

K9 glass is a high-quality borosilicate material characterized by several superior properties, including high optical transmittance, low refractive index, elevated hardness, a low thermal expansion coefficient, and excellent thermal stability [1,2]. These attributes facilitate effective control of light scattering, thereby significantly reducing aberrations within optical systems. Consequently, K9 glass is particularly well-suited for the fabrication of precision optical components. With the rapid advancement of optical and microsystem technologies, the cost-effectiveness and exceptional microfabrication capabilities of K9 glass have led to its widespread application in the production of microstructures and microdevices at micrometer and nanometer scales [3,4,5]. Such applications are especially prominent in complex optical systems, encompassing lasers, microscopes, astronomical telescopes, microwave technologies, and industrial inspection instruments. High-end optical systems typically demand extremely high surface accuracy and minimal processing-induced damage layers [6]. Therefore, the implementation of ultra-smooth surface treatments on K9 glass is critically important for enhancing the functional performance of optical and microwave devices. The intrinsic brittleness and elevated hardness of K9 glass present considerable obstacles to ultra-precision machining [7]. Presently, chemical mechanical polishing (CMP) technology, which leverages the synergistic interaction between chemical reactions and mechanical abrasion to facilitate effective substrate polishing, offers a viable solution [8,9]. CMP not only efficiently removes surface defects from the substrate but also substantially minimizes internal damage. Owing to its capacity to optimize the balance between surface quality and processing efficiency, CMP has emerged as a critical technique for the production of high-quality, high-yield K9 glass components.

In the CMP process, the polishing slurry facilitates the removal of substrate material by inducing chemical reactions at the substrate surface while simultaneously exerting frictional forces [9]. The efficacy of the polishing slurry significantly influences both the surface roughness of the substrate and the material removal rate (MRR), thereby serving as a critical determinant of CMP efficiency. Typically, the polishing slurry comprises abrasive particles combined with chemical additives. Cerium oxide (CeO_2_), as an abrasive, is capable of engaging in specific chemical interactions with glass or silica surfaces, thereby enabling an enhanced MRR [10,11,12]. Consequently, CeO_2_ abrasives are extensively employed in the polishing of silica-based materials. Nonetheless, polishing slurry composed exclusively of CeO_2_ abrasives exhibits limitations in terms of processing efficiency and surface quality. This has motivated efforts to optimize their performance through the incorporation of mixed abrasives and chemical additives [13]. Hence, a systematic investigation into the application of CeO_2_-based polishing slurry for the precision polishing of K9 glass, alongside the exploration of strategies to augment their effectiveness, holds significant theoretical importance and practical relevance.

Currently, the typical formulation of CeO_2_-based polishing slurries generally comprises the following categories of components: (i) abrasives (e.g., CeO_2_, La_2_O_3_, SiO_2_); (ii) oxidants/reductants (e.g., H_2_O_2_ and other peroxides); (iii) complexing/corrosion-inhibiting agents (e.g., citric acid, amino acids); (iv) dispersants/stabilizers (e.g., polyacrylic acid, PEG); (v) surfactants; and (vi) acids/bases (e.g., NaOH, NH_4_OH) for regulating the chemical environment [14,15,16,17,18,19,20,21]. The synergistic interactions among these components govern the interfacial reaction kinetics, particle dispersion stability, and abrasive–substrate contact behavior, thereby directly determining the MRR and surface quality. Previous studies have demonstrated that performance optimization of CeO_2_-based slurries can be achieved primarily via abrasive hybridization and the introduction of chemical additives. For example, Zhao et al. reported a CeO_2_-LaOF mixed abrasive system incorporating sodium N-lauroyl sarcosinate (SNLS) and sodium polyacrylate (PAAS) as dispersants [13]. Under alkaline conditions, this slurry exhibited significantly enhanced MRR and nanoscale surface roughness in quartz glass polishing, indicating that rare-earth oxyfluorides can effectively modulate the surface reactivity of CeO_2_ abrasives. Lv et al. formulated a polishing slurry consisting of potassium oleate (KOL), deionized water, CeO_2_, and molybdenum disulfide (MoS_2_) [22]. In this formulation, KOL functioned as both a surfactant and a pH regulator, thereby modulating the MRR of CeO_2_. Concurrently, MoS_2_ served as a solid lubricant, mitigating excessive mechanical damage to the substrate during the polishing process. Optimal concentrations of 0.2% KOL and 0.3% MoS_2_ in the polishing slurry yielded a quartz glass surface roughness of 0.48 nm and an MRR of 24.03 μm/h. He et al. investigated CeO_2_-SiO_2_ composite abrasives for K9 glass polishing and found that introducing 0.5 wt% SiO_2_ yielded the highest MRR (22.6 nm/min) and optimal surface roughness (1.3157 nm), highlighting the potential of composite abrasives for performance enhancement [23]. Nevertheless, the overall efficiency and surface precision remain insufficient for high-end applications. In addition to adding abrasives, appropriately formulated chemical additives also served a critical function. Polymer dispersants and small-molecule chelating agents (e.g., polyacrylates, carboxylates) have been shown to significantly improve the dispersion of CeO_2_ particles, modulate particle/substrate surface charge distribution, and reshape interfacial reaction pathways, thereby enhancing polishing uniformity and selectivity [24,25]. Moreover, the pH of the slurry significantly influenced the interaction between CeO_2_ and the substrate. It modulated the surface charge characteristics of both the particles and the substrate, thereby affecting their adsorption and reaction dynamics [26]. These factors subsequently influenced the formation and dissociation kinetics of transient Ce-O-Si bonds, thereby affecting both the efficiency of material removal and the extent of subsurface damage. Consequently, the combined modulation of pH and chemical additives has been extensively acknowledged as a critical approach for enhancing the performance of CeO_2_-based polishing slurries.

Despite prior research exploring the optimization of CeO_2_-based polishing slurries through the combination of mixed abrasives and chemical additives, challenges persist in achieving an optimal balance between MRR and surface quality in practical applications. Certain systems achieved relatively high removal efficiency but failed to adequately suppress surface damage and roughness, whereas others exhibited superior surface planarity at the expense of processing speed, limiting their suitability for large-scale, high-precision optical manufacturing. Therefore, striking an optimal balance between efficient MRR and high-quality surface finishing continues to be a central challenge for CeO_2_-based CMP processes. To address this issue, the present study proposed a novel slurry design strategy by incorporating Al_2_O_3_ into conventional CeO_2_ abrasives. It was expected to utilize the high chemical reactivity of CeO_2_ and the high hardness of Al_2_O_3_ in a synergistic manner to achieve an effective combination of chemical softening and mechanical wear, thereby achieving a balance between efficiency and precision. In terms of chemical additives, sodium laureth-6-carboxylate (SL-6C) and guanidine carbonate (GC) were introduced to improve particle dispersion and interfacial chemical reactivity. The SL-6C was capable of modifying the surface charge of abrasive particles, thereby improving the dispersion stability of the abrasive in the polishing slurry. Concurrently, the presence of GC reinforced the alkaline conditions, which facilitated the development of a softening layer on the glass surface. Herein, we aimed to develop a CeO_2_-Al_2_O_3_ mixed slurry capable of simultaneously achieving high MRR and superior surface quality, and to systematically evaluate its performance in the ultraprecision polishing of K9 glass. This work highlighted the synergistic mechanisms between mixed abrasives and the co-regulation of chemical additives, thereby offering not only a promising strategy for high-precision optical fabrication but also a new paradigm for the further optimization of CeO_2_-based CMP slurries.

## 2. Results and Discussion

### 2.1. Analysis of Abrasive Morphology and Evaluation of CMP Efficiency

**Figure 1:**
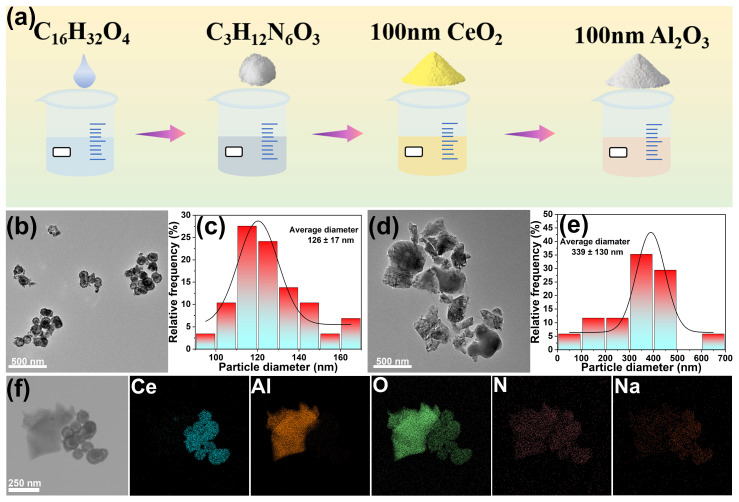
(**a**) Diagrammatic representation of the synthesis process of the polishing slurry, (**b**) TEM images of CeO_2_, (**c**) Size distribution of CeO_2_, (**d**) TEM images of Al_2_O_3_, (**e**) Size distribution of Al_2_O_3_, (**f**) EDS images of CeO_2_-Al_2_O_3_ mixed abrasive.

Figure 1a illustrated the schematic representation of the synthesis process for the polishing slurry. Sodium laureth-6-carboxylate (SL-6C), guanidine carbonate (GC), cerium oxide (CeO_2_), and alumina (Al_2_O_3_) were sequentially introduced into deionized water and subjected to ultrasonic dispersion to ensure homogeneous distribution. The morphology of the abrasive particles was examined using transmission electron microscopy (TEM). As shown in Figure 1b–e, the CeO_2_ particles had a spherical morphology with an average size of 126 ± 17 nm. The Al_2_O_3_ particles had an irregular shape with distinct edges, which was beneficial for increasing the polishing rate. However, the size distribution of the Al_2_O_3_ particles was wide, with an average size of approximately 339 ± 130 nm. Therefore, it was not advisable to introduce too many Al_2_O_3_ particles into the polishing slurry to avoid affecting the uniformity of the abrasive distribution in the solution [27]. Upon dispersion of the two abrasives within the polishing slurry, nitrogen (N) and sodium (Na) elements were found to be uniformly distributed across the surfaces of both abrasives, indicating effective adherence of the additives to the abrasive surfaces (Figure 1f).

**Figure 2:**
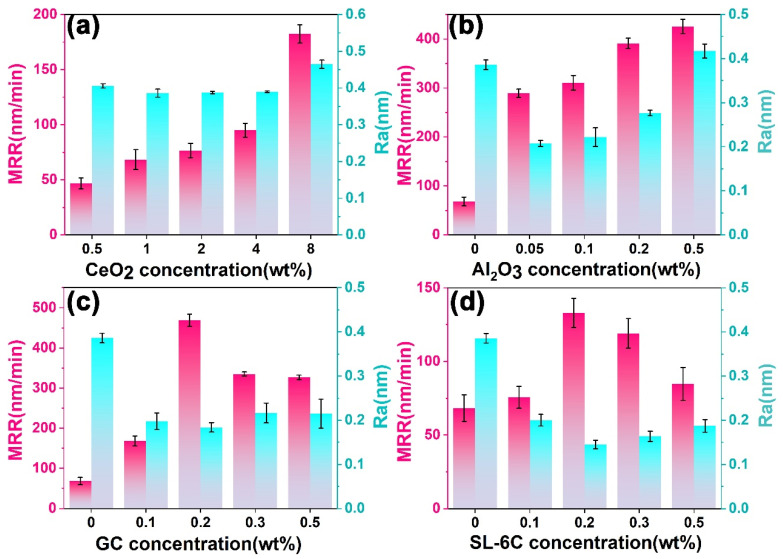
Screening of optimal polishing slurry formulation incorporation (**a**) CeO_2_, (**b**) Al_2_O_3_, (**c**) GC and (**d**) SL-6C.

To systematically evaluate the influence of individual components on polishing performance, a baseline slurry was prepared by dispersing CeO_2_ abrasives in deionized water and adjusting the pH to 10 using NaOH or acetic acid. A single-variable approach was then employed to determine the optimal concentrations of CeO_2_, Al_2_O_3_, GC, and SL-6C, using the material removal rate (MRR) and surface roughness (Ra) of K9 glass as the primary evaluation metrics. As shown in Figure 2a, increasing the CeO_2_ concentration from 0.5 wt% to 8 wt% resulted in a general rise in MRR, while Ra initially decreased slightly and then stabilized. Specifically, at 0.5 wt% CeO_2_, the MRR was only 46.70 nm/min with an Ra of 0.41 nm, whereas at 1 wt% CeO_2_ the MRR increased markedly to 68.33 nm/min and Ra decreased to 0.38 nm, indicating a favorable balance between MRR and surface finish. Further increasing to 2 wt% and 4 wt% CeO_2_ led to MRR values of 76.63 and 95.04 nm/min, respectively, with Ra remaining at approximately 0.39 nm. However, at 8 wt% CeO_2_, the MRR surged to 182.31 nm/min, but Ra increased to 0.46 nm, suggesting that excessive abrasive loading might promote particle agglomeration and induce micro-scratching, thereby degrading the surface quality. Taking into account the MRR, Ra values and cost issues, the optimal concentration of CeO_2_ abrasive was determined to be 1 wt%. Subsequently, we used a 1 wt% CeO_2_ solution as the base polishing slurry and conducted a screening of the amount of Al_2_O_3_. As shown in Figure 2b, increasing the Al_2_O_3_ concentration from 0.05 wt% to 0.5 wt% produced a general upward trend in MRR, while Ra initially decreased and then slightly rebounded. An excessive concentration of Al_2_O_3_ could lead to the aggregation of abrasive particles, which might induce localized micro-damage on the glass surface. This phenomenon adversely impacted the overall surface quality of the glass. Conversely, at an Al_2_O_3_ concentration of 0.05 wt%, the slurry demonstrated an optimal balance, achieving a high MRR while effectively reducing Ra. Consequently, this concentration was identified as the optimal Al_2_O_3_ content. Figure 2c,d illustrated the effects of GC and SL-6C concentrations on the MRR and Ra of K9 glass. GC was a strong base, which helped increase the alkalinity of the polishing slurry. This was beneficial for forming a softer and more elastic hydrated layer on the glass surface [28,29]. At an additive concentration of 0.2 wt% GC, the MRR reached its maximum value while concurrently maintaining the lowest Ra. However, increasing the concentration beyond this point led to a reduction in MRR and a marginal increase in Ra. This phenomenon might be attributed to excessive alkalinity, which potentially weakened the dispersion of abrasive particles, diminished chemical reactivity, or disrupted the synergistic interaction between chemical and mechanical processes at the interface of the abrasive particles and the substrate. SL-6C was an anionic surfactant characterized by the presence of carboxyl functional groups. It modulated the surface charge of abrasive particles via electrostatic and coordination interactions, thereby improving particle dispersion and mitigating agglomeration. This in turn minimized scratching risk while improving interfacial lubrication and lowering the detachment energy of the reaction layer. At an SL-6C concentration of 0.2 wt%, the polishing slurry attained its highest MRR and the lowest Ra. Increasing the concentration beyond this point might lead to excessive surfactant adsorption or over-lubrication, which could diminish the abrasive’s cutting effectiveness and consequently decrease the MRR. Integrating the findings from Figure 2a–d, the optimal polishing slurry was established as follows: 1 wt% CeO_2_ as the primary abrasive, 0.05 wt% Al_2_O_3_ as the secondary abrasive, 0.2 wt% GC and 0.2 wt% SL-6C as chemical additives, and pH was adjusted to 10. This formulation concurrently improved the dispersion stability of the particles, the interfacial lubricity, and the chemical reactivity of CeO_2_, resulting in a substantial increase in MRR and a marked reduction in Ra. The synergistic interaction between chemical corrosion and mechanical wear under these conditions underscored the critical importance of the coordinated selection of abrasives and chemical additives to optimize the performance of CeO_2_-based CMP slurries.

**Figure 3:**
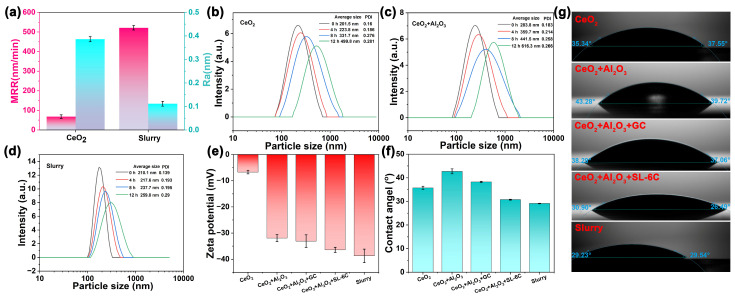
Comparison for baseline slurry and optimized slurry in (**a**) MRR and Ra, DLS analysis of (**b**) baseline slurry, (**c**) Adding 0.05 wt% Al_2_O_3_ to the baseline slurry and (**d**) optimized slurry, (**e**) Zeta potential of different slurries, *n* = 3, (**f**) Contact angle of different slurries, *n* = 3, (**g**) Real-time contact angle images.

As illustrated in Figure 3a, the base polishing slurry employed consisted of a CeO_2_ suspension with a mass fraction of 1 wt% and a pH value of 10. A marked difference in performance was observed between this basic slurry and the optimized polishing slurry developed in the present study. Specifically, the MRR of the basic polishing slurry was relatively low, measured at approximately 68.33 nm/min, with an Ra of 0.38 nm. In contrast, the optimized slurry exhibited a substantially enhanced MRR of 521.71 nm/min, accompanied by a reduced Ra value of 0.11 nm. These results demonstrated that the optimized polishing slurry could simultaneously achieve high MRR and superior surface quality. Dynamic light scattering (DLS) analysis was utilized to evaluate the dispersion stability of the polishing slurry. This technique involves monitoring the intensity of scattered light and its temporal variations to derive kinetic information about the powder particles, which is subsequently used to calculate the particle size distribution. It is important to note that DLS measures the hydrated particle size within the solution rather than the actual physical size. For non-spherical particles, the hydrated size is estimated based on an equivalent volume diameter, which limits the accuracy of size determination for irregularly shaped nanomaterials. Thus, DLS primarily provides relative size ranges and trends when comparing similar materials. Consequently, electron microscopy is typically employed to ascertain the true particle size, while DLS offers insight into the overall particle size distribution within the sample. The DLS results revealed that the hydrated particle size of CeO_2_ in the pure CeO_2_ polishing slurry was 201.5 nm (Figure 3b). Over time, an increase in hydrated particle size was observed, indicating poor dispersion stability of the pure CeO_2_ slurry. Upon the addition of 0.05 wt% Al_2_O_3_ nanoparticles, the hydrated particle size of the mixed abrasive increased to 283.8 nm, suggesting that the incorporation of larger Al_2_O_3_ nanoparticles significantly enlarged the overall particle size (Figure 3c). Furthermore, the hydrated particle size continued to increase with storage time, implying that the polishing slurry containing only CeO_2_ and Al_2_O_3_ abrasives exhibited instability, with a tendency for particle agglomeration and sedimentation. In the optimized polishing slurry, DLS measurements primarily served to assess the overall dispersion state of the CeO_2_-Al_2_O_3_ mixed abrasives rather than to determine the precise size of individual particles. The measured hydrated particle size represented a weighted average reflecting the combined effects of both components. Complementary EDS analysis (Figure 1f) demonstrated that during slurry preparation, GC and SL-6C additives were uniformly adsorbed onto the abrasive surfaces, facilitating improved dispersion. As depicted in Figure 3d, the particle size of the mixed abrasive in the optimized polishing slurry was 210.1 nm, indicating that despite the large and irregular morphology of Al_2_O_3_ particles, the additives effectively enhanced abrasive dispersion. Over extended reaction times, only a slight increase in hydrated particle size was observed, signifying that the inclusion of GC and SL-6C markedly improved the dispersion stability of the polishing slurry. We further conducted Zeta potential and contact angle tests, as illustrated in Figure 3e,f. The optimized polishing slurry formulation resulted in an increase in the surface potential of the particles from −6.73 mV to −38.7 mV, accompanied by a decrease in the contact angle from 37.72 ± 0.71° to 29.11 ± 0.11°. These findings suggested that the incorporation of additives significantly enhanced both the wetting characteristics and the dispersion stability of the polishing slurry. The enhancement in surface wettability was anticipated to facilitate more efficient and uniform interactions between the particles and the substrate [22]. Furthermore, the real-time contact angle image depicted in Figure 3g demonstrated that the liquid film formed by the optimized polishing slurry on the glass surface exhibited a more homogeneous distribution, thereby providing direct evidence of the improved wetting properties.

Previous studies have shown that by using abrasive mixtures and adding chemical additives, the performance of abrasive slurry based on CeO_2_ can be optimized. In the mixed abrasive polishing slurry, for the selection of the second type of abrasive, there were generally three approaches. The first was to choose a second type of abrasive with higher hardness, using the high hardness of the second abrasive to significantly increase the removal rate of cerium-based abrasive [19]. However, this situation might lead to poor surface quality of the wafer after polishing. The second was to add two-dimensional materials as solid lubricants [20]. The addition of solid lubricants could reduce the excessive friction between CeO_2_ and the workpiece, thereby improving the surface quality of the polished wafer. The third was to add lanthanide metal oxides [21]. During the polishing process, the lanthanide metal oxides might enhance the chemical activity of CeO_2_, thereby improving its polishing performance. For the selection of additives, generally, anionic-type surfactants were chosen. This type of surfactant not only improved the dispersion of CeO_2_ but also helped to enhance the chemical activity of CeO_2_, thereby improving the polishing performance of cerium-based abrasive. As summarized in Table 1, by comparing representative works with our own work, it could be seen that this polishing slurry formula led in both cases to the removal rate of K9 glass and the surface quality of K9 glass after polishing. These comparisons clearly demonstrated the superior polishing performance and practical potential of the designed slurry formulation.

**Figure 4:**
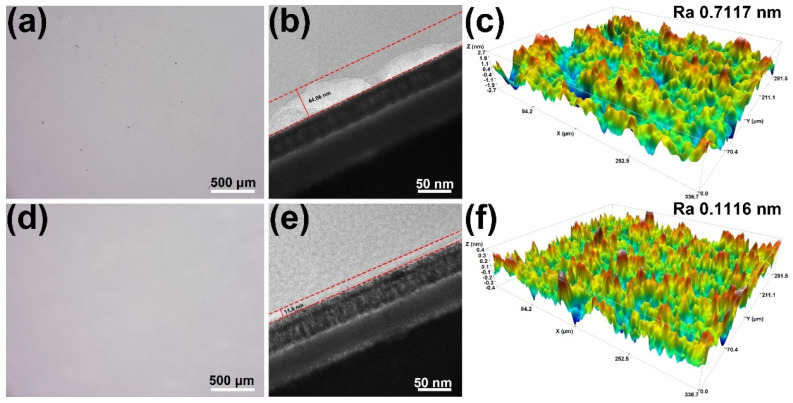
Ultra-depth-of-field microscope images of (**a**) original surface of K9 glass and (**d**) polished surface using optimized slurry; TEM images of the surface damage layer of (**b**) the original glass and (**e**) polished surface using optimized slurry; 3D surface profile of (**c**) the original glass surface and (**f**) polished surface using optimized slurry.

Ultra-depth-of-field optical microscopy was employed to systematically examine the surface morphology of K9 glass before and after polishing (Figure 4a,d). Prior to polishing, the glass surface exhibited pronounced scratches, pits, and other irregular defects, indicative of severe processing-induced damage. After treatment with the optimized slurry, the K9 glass surface became markedly flatter, smoother, and more continuous, with virtually no discernible remnants of the original defects. This direct visual evidence highlighted the superior capability of the formulated slurry to remove surface damage and improve micro-scale flatness. Figure 4b,e further illustrated the subsurface damage state of the K9 glass substrates before and after polishing. Before polishing, a distinct damage layer with a thickness of approximately 44.06 nm was present, reflecting structural disruption introduced during prior machining. After polishing with the optimized slurry, the damage layer thickness was significantly reduced to 11.8 nm, suggesting that the slurry effectively removed surface defects. Figure 4c,f showed the three-dimensional (3D) surface topographies of K9 glass before and after polishing. Prior to polishing, the surface of the K9 glass exhibited pronounced undulations and a relatively high Ra value of 0.7117 nm, characterized by numerous protrusions and micro-cracks. In contrast, following treatment with the polishing slurry, the surface roughness significantly decreased to 0.1116 nm, and the surface morphology became notably smoother and more uniform.

### 2.2. Analysis of CMP Mechanism

**Figure 5:**
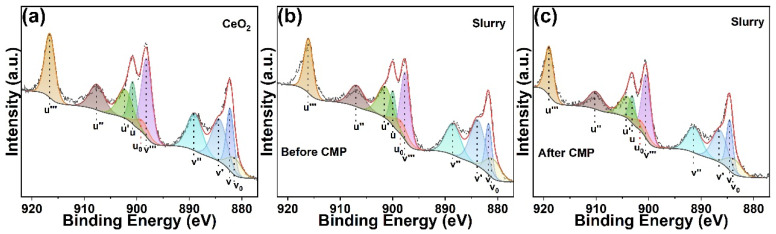
Ce 3d XPS spectra of (**a**) CeO_2_, (**b**) Slurry before polishing and (**c**) Slurry after polishing.

XPS tests were conducted on pure CeO_2_ and the polishing slurry before and after the polishing process. The Ce 3d spectrum was decomposed into ten distinct peaks (Figure 5a–c). Specifically, the peaks labeled as v, v″, v‴, u, u″ and u‴ corresponded to the Ce^4+^ species, while v_0_, v’, u_0_ and u’ were classified as the Ce^3+^ species [30,31]. The semi-quantitative analysis based on peak area fitting was used to determine the concentration of Ce^3+^ ions. The concentration of Ce^3+^ ions on the surface of pure CeO_2_ was measured at 31.48%. Following the preparation of the polishing slurry, the Ce^3+^ ion concentration on the CeO_2_ surface increased markedly to 40.37%, suggesting that the incorporation of Al_2_O_3_, SL-6C, and GC substantially enhanced the chemical reactivity of CeO_2_. Subsequent to the polishing process using this slurry, the Ce^3+^ concentration on the CeO_2_ surface decreased to 35.88%. This reduction was likely attributable to the consumption of Ce^3+^ ions during the polishing process.

**Figure 6:**
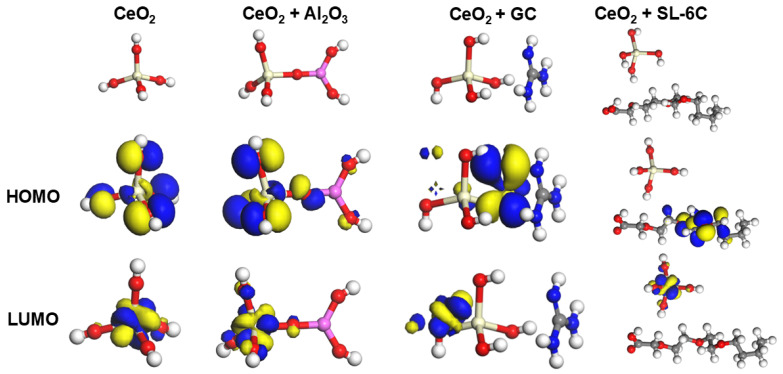
HUMO and LUMO of CeO_2_, CeO_2_ + Al_2_O_3_, CeO_2_ + GC, and CeO_2_ + SL-6C.

We further explored the interactions between Al_2_O_3_, GC, SL-6C and CeO_2_ through theoretical calculations. According to the Frontier Molecular Orbital (FMO) calculations, the highest occupied molecular orbital (HOMO) exhibited a lower electron binding energy, indicating its donor characteristics, while the lowest unoccupied molecular orbital (LUMO) showed significant electron affinity, consistent with the receptor behavior [32,33,34]. As shown in Figure 6, for pure CeO_2_, the HOMO (−8.151 eV) of a single repeating unit Ce(OH)_4_ was uniformly distributed throughout the molecule, while its LUMO (−6.049 eV) was located on the Ce atom, forming a HOMO-LUMO gap of 2.102 eV. When Al_2_O_3_ was introduced into the Ce(OH)_4_ structure, the gap between HOMO (−6.586 eV) and LUMO (−4.8651 eV) decreased to 1.735 eV, indicating that electron transfer occurred between CeO_2_ and Al_2_O_3_, and the chemical activity of the CeO_2_-Al_2_O_3_ mixed abrasive was higher [35]. In the CeO_2_ + GC system, the HOMO (−6.586 eV) was mainly distributed in GC, while the LUMO (−4.851 eV) was mainly distributed in CeO_2_, and the HUMO-LUMO gap (1.459 eV) was further reduced, indicating that the chemical activity between CeO_2_ + GC was also significantly enhanced. In the CeO_2_ + SL-6C system, its HUMO (−6.646 eV) was mainly distributed in SL-6C, while LUMO (−4.797 eV) was mainly distributed in CeO_2_, and the HUMO-LUMO gap (1.849 eV) was also lower than that of CeO_2_, indicating that the addition of SL-6C also significantly affected the chemical activity of CeO_2_. Theoretical calculations further supported the CMP experiment. We further calculated the changes in surface energy of each component after mixing with cerium oxide, as shown in Table 2. The surface energy of pure CeO_2_ was −36.9 kJ/A^2^. When GC, SL-6C, and Al_2_O_3_ were added, the surface energy gradually decreased to −37.9 kJ/A^2^, −40.7 kJ/A^2^, and −37.6 kJ/A^2^, indicating that the addition of all three materials could enhance the dispersion stability of CeO_2_ [36,37]. Particularly, the addition of SL-6C reduced its surface energy, suggesting that SL-6C, as a surfactant, had the best effect in improving the dispersion stability of CeO_2_.

**Figure 7:**
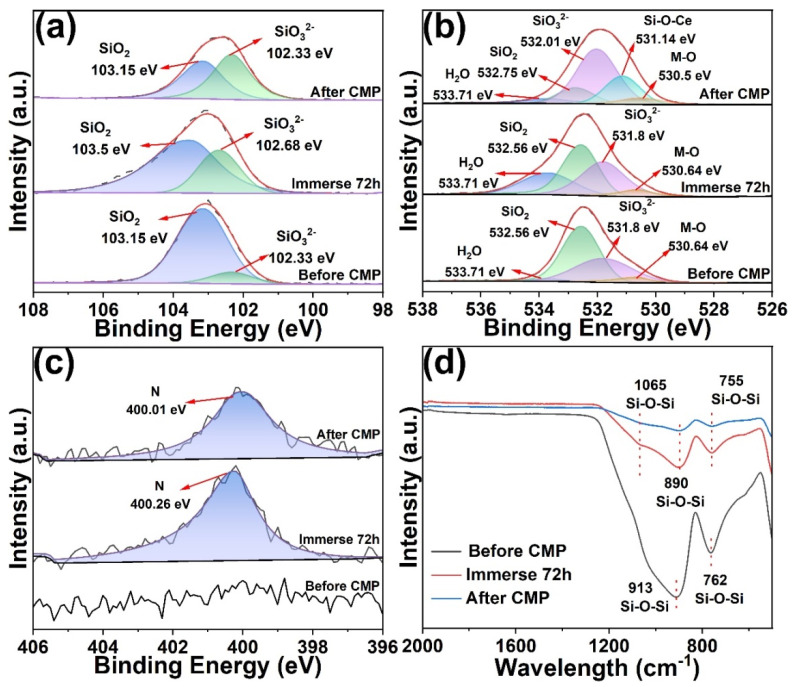
XPS spectra of K9 glass surface before and after CMP, and after immersion in the optimized slurry for 72 h: (**a**) Si 2p spectra, (**b**) O 1s spectra, (**c**) N 1s spectra. (**d**) FTIR spectra of K9 glass surfaces before and after CMP, and after immersion in the optimized slurry for 72 h.

To elucidate the role of the optimized slurry formulation in surface chemical modification and material removal on K9 glass, XPS and FTIR analyses were performed on the substrates before polishing, after immersion in the optimized slurry for 72 h, and after polishing (Figure 7). The Si 2p spectra showed that the unpolished sample exhibited a dominant SiO_2_ peak at 103.15 eV and only a weak SiO_3_^2−^ signal at 102.33 eV (Figure 7a) [38,39]. After 72 h immersion, the SiO_3_^2−^ signal became markedly stronger, approaching but still slightly lower than that of SiO_2_, indicating that under alkaline conditions the slurry induced hydration/depolymerization of the glass surface, thereby generating a higher fraction of low-polymerized silicate species. After polishing, the SiO_3_^2−^ component further increased and surpassed the SiO_2_ peak, suggesting that chemical–mechanical coupling produced a depolymerized, silicate-rich layer that was more easily removed. Complementary evidence was provided by deconvolution of the O 1s spectra (Figure 7b). Before polishing, distinct peaks were observed at 533.71 eV (adsorbed H_2_O), 532.56 eV (SiO_2_), and 531.8 eV (SiO_3_^2−^), while the metal oxide (M-O) contribution at 530.64 eV was minimal [40]. After 72 h immersion, the adsorbed water and SiO_3_^2−^ components increased while the SiO_2_ component slightly decreased, confirming that hydration facilitated partial cleavage of the Si-O-Si network and formation of hydroxylated surfaces. Critically, after polishing, SiO_3_^2−^ became the dominant O 1s component, and a new peak appeared at 531.14 eV attributable to a Si-O-Ce chemical state, implying that CeO_2_ interacted chemically with the glass surface through transient Ce-O-Si bonding [21,40]. This interaction lowered the binding strength of the reaction layer and enhanced its susceptibility to shear removal. In contrast, the M-O signal remained essentially unchanged across all three states, suggesting that bulk metal oxide content was not fundamentally altered or massively introduced. The N 1s spectra (Figure 7c) showed only background noise before polishing but a distinct peak at 400 eV after immersion and polishing, indicating adsorption or incorporation of N species (derived from GC) into the surface/reaction layer. These nitrogen-containing species might adjust surface charge and local chemistry through weak coordination or electrostatic interactions, thereby facilitating Ce-O-Si bond formation and accelerating interfacial reactions, consistent with the observed changes in Zeta potential and contact angle. FTIR spectra further corroborated these findings (Figure 7d). The unpolished sample displayed characteristic Si-O-Si stretching bands at 913 and 762 cm^−1^ [41,42,43]. After 72 h of immersion, the intensities of these SiO_2_-related peaks decreased and exhibited slight shifts. After polishing, the peaks weakened and shifted further, revealing hydrolysis/cleavage and chemical modification of the Si-O-Si framework. This attenuation could be ascribed to partial Si-O bond breakage and new bonding with Ce or additives (e.g., Ce-O-Si), thereby diminishing the original Si-O-Si vibrational response.

**Figure 8:**
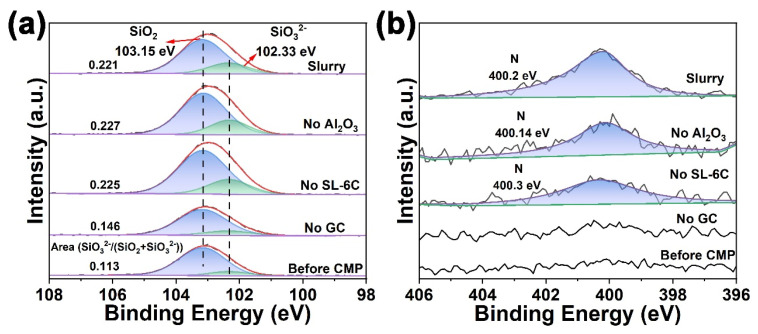
XPS spectra of K9 glass surface after immersion in the different slurries for 24 h: (**a**) Si 2p spectra, (**b**) N 1s spectra.

An additional experiment was conducted in which glass sheets were immersed in the polishing slurry for a duration of 24 h. A comparative analysis of Figure 7a and Figure 8a revealed that prolonging the immersion time resulted in an increased presence of the hydrated layer on the glass surface. Subsequently, the influence of specific additives within the polishing slurry on the formation of the hydrated layer during immersion was examined. Glass sheets were immersed for 24 h in slurries lacking GC, SL-6C, and Al_2_O_3_, respectively, followed by surface analysis using XPS. Analysis of the Si 2p spectra indicated that the absence of GC in the slurry corresponded with a markedly diminished SiO_3_^2−^ signal on the glass surface compared to samples containing GC, suggesting that the inclusion of GC facilitated the development of a softening layer on the glass surface (Figure 8a). Furthermore, the presence of the N 1s signal on the K9 glass surface was detected only after the addition of the GC additive to the polishing slurry (Figure 8b).

**Figure 9:**
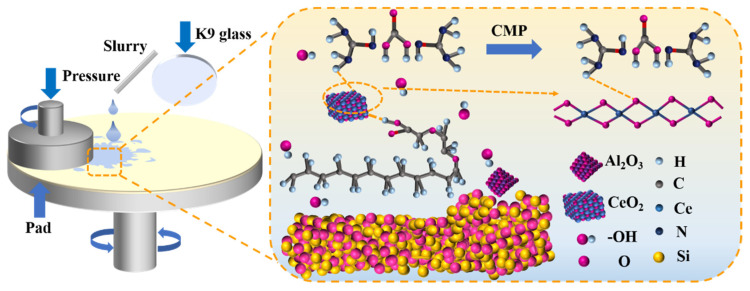
Schematic diagram of the polishing mechanism of polishing slurry.

Taken together, the systematic XPS and FTIR results demonstrated that, under the combined action of alkaline slurry and chemical additives, the K9 glass surface first underwent hydration/depolymerization to form a soft, SiO_3_^2−^-rich reaction layer [44,45]. CeO_2_ then chemically interacted with this layer, producing transient Si-O-Ce bonds that weaken interfacial binding and render the layer more easily sheared away by abrasives (Figure 9). The intermediate state observed after immersion (enhanced H_2_O and SiO_3_^2−^ signals, emergence of N 1s) supported a two-step mechanism of chemical activation followed by mechanical removal. This mechanistic insight explains why the optimized formulation not only dramatically increases MRR but also minimizes subsurface damage and improves surface planarity, achieving high polishing efficiency and superior surface quality through synergistic chemical softening and mechanical removal.

## 3. Materials and Methods

### 3.1. Materials

All the materials were purchased from Xilong Chemical and did not undergo any further purification.

### 3.2. Preparation of CeO_2_ Abrasive and Slurry

A homogeneous solution was prepared by dissolving 17.5 g cerium nitrate hexahydrate (Ce(NO_3_)_3_·6H_2_O), 3.95 g hexamethylenetetramine (HMT, C_6_H_12_N_4_), and 1 g tartaric acid (C_4_H_6_O_6_) in 5000 mL deionized water under ultrasonic dispersion for 10 min. The resulting solution was subsequently heated to 80 °C and maintained at this temperature for 8 h under continuous magnetic stirring (500 rpm). The precipitated product was isolated via centrifugation (10,000 rpm, 3 min) and subjected to sequential washing cycles using deionized water and anhydrous ethanol (3 times each) to remove residual ionic impurities. After vacuum drying at 60 °C for 24 h, the cerium-based precursor was transferred to a tubular furnace for calcination. The thermal treatment involved a controlled heating rate of 5 °C/min up to 800 °C, with a dwell time of 4 h under ambient atmosphere, yielding CeO_2_ abrasive.

Sodium laureth-6-carboxylate, guanidine carbonate, Al_2_O_3_, and CeO_2_ abrasives were combined in specific proportions with deionized water. Subsequently, the pH value of the above solution was tested using a pH meter. If the pH value was higher than 10, the pH of the solution was adjusted to 10 using acetic acid. If the pH value was lower than 10, the pH was adjustedto 10 using sodium hydroxide. Then, the prepared solution was subjected to ultrasonic treatment at ambient temperature for 1 h to produce a homogeneous suspension.

### 3.3. Characterization

High-resolution transmission electron microscopy (HR-TEM, JEM ARM-200F) was employed to characterize the morphology of the materials. The hydrodynamic particle size distribution and zeta potential were determined with a dynamic light scattering analyzer (ELSZ-2000ZS, Otsuka Electronics, Suzhou, China). The elemental composition and chemical states were examined by X-ray photoelectron spectroscopy (XPS, AXIS Supra, Shimadzu, Kyoto, Japan). During the XPS data processing procedure, the background subtraction method adopted was the Smart method, with the FWHM range set at 0.5–3.5 eV. The fitting constraints were fixed as 30% for the Lorentz/Gaussian mixture, 100% for the tail mixture, 0% for the tail height, and 0% for the tail index. The reference position was obtained by performing peak-fitting using the spectra of standard Ce^4+^ and Ce^3+^. The distances between the Ce^4+^ peaks and the distances between the Ce^3+^ peaks were calculated, and then peak-fitting was carried out using the bimodal peak-fitting mode to maintain the distances between the peaks as much as possible without changing them. The charge reference was calibrated using the C 1s at 284.8 eV, as the main purpose was to investigate the activity changes in the Ce element before and after polishing. The analysis of the total spectrum was not very significant for this.

### 3.4. CMP Test

K9 glass substrates (8-inch) were obtained from Qingdao Mingyu Technology Co., Ltd., (Qingdao, China). For comparative purposes, commercial Al_2_O_3_ abrasives with an average particle size of 100 nm were purchased from Zibo Xiyan Nanomaterials Co., Ltd., (Zibo, China). Polishing experiments were conducted on a polishing machine (Shenzhen Fangda Grinding Technology Co., Ltd., Shenzhen, China) equipped with a Suba 800 polishing pad (Dupont, Wilmington, DE, USA). The operating conditions were set as follows: platen rotation speed, 55 rpm; polishing head rotation speed, 55 rpm; applied weight, 80 kg. Then the load was 784 N, according to the formula P = F/S (where P represents pressure, F represents load, and S represents area), and it could be known that the pressure applied to the surface of the glass was 24.95 kPa; polishing duration, 2 min; and slurry feed rate, 50 mL/min.

After polishing, the K9 glass samples were repeatedly rinsed in deionized water and dried using an ultrasonic cleaner. The surface roughness was measured using a three-dimensional surface profilometer (Sneox 090, SENOFAR, Barcelona, Spain) over a scanning area of 336.71 × 281.51 µm^2^. The sample weight was recorded using a precision electronic balance with an accuracy of 0.1 mg. The polishing efficiency, expressed as the material removal rate (MRR, nm/min), was calculated according to Equation (1).

(1) $$\mathrm{MRR}=\frac{M_{0}-M}{\rho \times S\times t}$$

Here, M_0_ represented the quality of K9 glass before polishing, M represented the quality of K9 glass after polishing, ρ represented the density of K9 glass (2.53 g/cm^3^), S represented the area of K9 glass (314.2 cm^2^), and t represented the polishing time (2 min).

## 4. Conclusions

This study tackled the long-standing challenge of simultaneously achieving a high MRR and superior surface quality in CeO_2_-based polishing slurries by introducing a novel slurry design strategy that integrates CeO_2_-Al_2_O_3_ mixed abrasives with synergistic chemical additives. Leveraging the pronounced chemical reactivity of CeO_2_, robust transient Ce-O-Si bonds were established with the surface of K9 glass. Furthermore, owing to its elevated hardness and superior mechanical wear resistance, Al_2_O_3_ contributed to improving the MRR while preserving surface flatness. Through the co-integration of the two abrasives and precise pH adjustment to 10, the slurry established an effective synergy between chemical softening and mechanical stripping, thereby overcoming the intrinsic trade-off between MRR and Ra inherent to conventional single-abrasive CeO_2_ slurries. Performance evaluations demonstrated that the optimized composite-abrasive slurry achieves nearly a seven-fold increase in MRR and a 71% reduction in Ra compared with the baseline slurry, highlighting the pronounced benefits of coupling composite abrasives with tailored chemical additives. Complementary Zeta potential, contact angle, and DLS analyses confirmed that this formulation markedly enhanced particle surface potential, dispersion stability, and wettability, facilitating uniform adsorption and interfacial interactions of abrasives on the glass surface and thereby establishing favorable conditions for efficient CMP. XPS and FTIR analyses further elucidated the underlying chemo-mechanical synergy. Under alkaline conditions and in the presence of synergistic additives, the glass surface undergoes hydration and depolymerization to form a SiO_3_^2−^-rich softened layer. Subsequent action of the CeO_2_-Al_2_O_3_ mixed abrasives enabled dual chemical–mechanical removal: CeO_2_ lowered interfacial binding energy by forming transient Si-O-Ce linkages, while Al_2_O_3_ enhanced shear and stripping capability, collectively promoting efficient detachment of the reaction layer. Attenuation and shifts in FTIR vibrational bands corroborated the disruption of the Si-O-Si framework and the formation of new chemical bonds such as Si-O-Ce. In summary, this work demonstrated and validated a new CMP slurry design concept based on the synergistic integration of CeO_2_-Al_2_O_3_ mixed abrasives, targeted chemical additives. This approach not only delivered a substantial increase in MRR but also ensured excellent surface integrity during ultra-precision polishing. By effectively coupling chemical softening with mechanical abrasion, the proposed slurry provided a robust and scalable pathway for high-efficiency, low-damage processing of hard and brittle optical materials. Beyond offering a viable paradigm for the precision manufacturing of optical components and semiconductor substrates, these findings also furnished new insights into mixed abrasive engineering and interfacial chemical synergy, guiding future optimization of CeO_2_-based CMP systems.

## Figures and Tables

**Table 1:** Statistical data on the polishing effects of slurry with different abrasives and chemical additives.

Abrasives	Chemical Additives	Types of Glass	Ra (nm)	MRR (nm/min)	Ref.
CeO_2_ + LaOF	SNLS + PAAS	Quartz glass	0.23	530.52	[13]
CeO_2_ + diamond	/	Glass	0.6	109.6	[19]
CeO_2_ + h-BN	KOL	Fused silica	0.124	532	[20]
CeO_2_ + Er_2_O_3_	STPP + ChCl	Fused silica	0.07	625.5	[21]
CeO_2_ + MoS_2_	KOL	Quartz glass	0.48	400.5	[22]
CeO_2_ + Al_2_O_3_	GC + SL-6C	K9	0.11	521.71	This work

**Table 2:** HUMO, LUMO and surface energy of CeO_2_, CeO_2_ + Al_2_O_3_, CeO_2_ + GC, and CeO_2_ + SL-6C.

Structure	CeO_2_	CeO_2_ + Al_2_O_3_	CeO_2_ + GC	CeO_2_ + SL-6C
HUMO (eV)	−8.151	−6.586	−6.109	−6.646
LUMO (eV)	−6.049	−4.851	−4.650	−4.797
HOMO-LUMO gap (eV)	2.102	1.735	1.459	1.849
Surface energy (kJ/A^2^)	−36.9	−37.6	−37.9	−40.7

## Data Availability

The original contributions presented in this study are included in the articl. Further inquiries can be directed to the corresponding authors.
